# YOLO-DST: MEMS Small-Object Defect Detection Method Based on Dynamic Channel–Spatial Modeling and Multi-Attention Fusion

**DOI:** 10.3390/s26020369

**Published:** 2026-01-06

**Authors:** Qianwen Su, Hanshan Li

**Affiliations:** School of Electronic and Information Engineering, Xi’an Technological University, Xi’an 710021, China; qianwensu_2002@st.xatu.edu.cn

**Keywords:** MEMS defect detection, small-object detection, multi-attention fusion, triplet attention

## Abstract

During the process of defect detection in Micro-Electro-Mechanical Systems (MEMSs), there are many problems with the metallographic images, such as complex backgrounds, strong texture interference, and blurred defect edges. As a result, bond wire breaks and internal cavity contaminants are difficult to effectively identify, which seriously affects the reliability of the whole machine. To solve this problem, this paper proposes a MEMS small-object defect detection method, YOLO-DST (Dynamic Channel–Spatial Modeling and Triplet Attention-based YOLO), based on dynamic channel–spatial blocks and multi-attention fusion. Based on the YOLOv8s framework, the proposed method integrates dynamic channel–space blocks into the backbone and detection head to enhance feature representation across multiple defect scales. The neck of the network integrates multiple triple attention mechanisms, effectively suppressing the background interference caused by complex metallographic textures. Combined with the small-object perception enhancement network based on a Transformer, this method improves the capture ability and stability of the model for the detection of bond wire breaks and internal cavity contaminants. In the verification stage, a MEMS small-object defect dataset covering typical metallographic imaging was constructed. Through comparative experiments with the existing mainstream detection models, the results showed that YOLO-DST achieved better performance in indicators such as Precision and mAP@50%.

## 1. Introduction

Micro-assembly device substrates play a critical role in advanced manufacturing and high-end equipment systems by supporting various sensitive chips and micro-scale structures [1,2]. Among them, MEMS (Micro-Electro-Mechanical System) devices serve as essential functional units responsible for signal acquisition, state monitoring, and actuation, and their reliability directly determines the operational stability and safety of the entire system [3]. During the manufacturing process, the internal microstructures of uncapped MEMS devices remain fully exposed and are easily affected by process residues, mechanical disturbances, or particle contamination during the wafer fabrication, dicing, bonding, and assembly stages, which may lead to defects such as bond wire breaks and internal cavity contaminants [4,5]. These factors may introduce micro-scale defects such as bond-wire breakage and cavity impurities. Because such defects are extremely small and often visually inconspicuous, manual inspection struggles to identify them accurately in a timely manner [6]. If undetected before sealing, these defects may lead to performance degradation or even functional failure during subsequent packaging or using stages [7]. Therefore, developing an automated detection method capable of efficiently and accurately recognizing defects in uncovered MEMS devices is of great significance for enhancing the reliability of micro-assembly systems.

Defect detection of traditional MEMS devices mainly relies on the combination of automatic optical inspection (AOI) and manual re-inspection. Conventional defect inspection for MEMS devices typically relies on automatic optical inspection (AOI) combined with manual verification, yet this workflow remains insufficient when dealing with typical small-object defects such as bond wire breaks and internal cavity contaminants. The limitations originate from several aspects. First, AOI systems are primarily based on predefined rules or traditional image-processing algorithms, making them highly sensitive to the complex textures, illumination reflections, and background noise inherent in metallographic images. As a result, defects characterized by weak textures, extremely small sizes, or blurred edges are prone to being missed or misclassified [8]. Second, existing inspection pipelines largely focus on macroscopic surface anomalies and lack the ability to accurately model micro-scale structural details [9]. Under multi-light-source illumination, the exposed bonding wires and microcavity particles of uncovered MEMS devices often show non-uniform textures that are heavily mixed with the background, further complicating defect extraction. In addition, AOI results typically require secondary manual confirmation, which increases inspection cost and makes it difficult to maintain consistency and real-time performance, falling short of the requirements for pre-packaging online inspection of high-reliability MEMS devices. Therefore, achieving accurate detection of small-object defects such as bond wire breaks and internal cavity contaminants under complex metallographic backgrounds has become a critical challenge in current MEMS defect inspection research.

In recent years, deep learning has enabled automatic feature extraction and real-time inference, making it a predominant approach for defect detection in industrial manufacturing. Uncovered MEMS devices often contain two typical categories of small-object defects, namely bond wire breaks and internal cavity contaminants. These defects are characterized by extremely small sizes, blurred edges, weak textures, and a strong tendency to be overwhelmed by complex metallographic backgrounds, which makes their precise detection very challenging. To address these difficulties, this study develops a small-object defect detection network based on the YOLOv8s framework. The network incorporates the DCS block in both the backbone and detection head to enhance multi-scale feature representation, integrates the Triplet Attention mechanism into the neck to suppress background interference, and employs a Transformer-based small-object perception enhancement network to strengthen the capture of micro-scale defects under weak-texture conditions. This detection framework is designed to provide a reliable solution for small-object defect screening in uncovered MEMS devices.

The key contributions of this study can be summarized as follows:

- This study constructs a MEMS small-object defect dataset under representative metallographic imaging conditions and proposes a MEMS small-object defect detection method, YOLO-DST, which integrates Dynamic Channel–Spatial Modeling and multiple attention mechanisms. The method achieves highly accurate detection of micro-scale defects such as bond wire breaks and internal cavity contaminants, and provides an effective technical foundation for quality screening before device encapsulation.
- YOLO-DST incorporates the DCS Block into both the backbone and the detection head and integrates the Triplet Attention mechanism into the neck structure to strengthen the model’s multi-scale feature representation capability. These enhancements allow the network to better model defect characteristics at different scales and effectively suppress interference caused by complex metallographic textures.
- YOLO-DST integrates the small-object perception enhancement network to improve the model’s ability to capture extremely small defects under weak-texture and high-noise conditions. This enhancement enables the network to respond more sensitively to fine structural defects, including bond wire breaks and internal cavity contaminants, and significantly improves detection accuracy, robustness, and generalization performance, offering reliable support for the automated quality screening of uncovered MEMS devices.

## 2. Related Work

Early studies on small-object defect detection for micro-assembly substrates and micro-assembly components primarily relied on traditional machine vision and non-destructive testing (NDT) techniques. In electronic and integrated-circuit packaging, methods such as automated optical inspection (AOI), X-ray imaging, and acoustic microscopy have been widely applied to structural evaluation of solder voids, package cracking, and delamination; however, these approaches are highly sensitive to equipment cost, imaging conditions, and morphological variations in inspected structures, making it difficult to achieve both high detection accuracy and online inspection efficiency [10]. For finer-scale surface defects, Ng et al. introduced an automatic thresholding-based defect segmentation method that improves global threshold selection to enhance the separability of low-contrast defect regions, yet misclassification remains likely in scenarios with complex background textures or strong noise [11]. Beyond these works, several traditional digital image-processing approaches have also been explored in micro-assembly and MEMS-related defect inspection. Hou et al. employed differential imaging combined with mathematical morphology for IC wafer defect detection, but their method depends heavily on the availability of defect-free template images and shows limited robustness under unstable illumination or complex pattern conditions [12]. Liu et al. further proposed a two-dimensional wavelet-transform-based wafer defect detection algorithm that improves robustness to brightness variations by constructing multi-frame reference images, though its reliance on multiple acquisitions restricts its applicability to online inspection scenarios [13]. Qu et al. developed a directional morphological-gradient technique capable of distinguishing scratches, particles, and stains on wafer surfaces, yet its sensitivity to weak-texture and low-contrast defects remains limited [14]. Cheng et al. introduced a geometrical defect detection method for non-silicon MEMS components using Hu invariant moments extracted from skeleton images, but the method struggles with metallographic noise and subtle micro-cracks that deviate from ideal geometric patterns [15]. In micro-device assembly tasks, Baidyk et al. used flat-image-based pattern recognition to aid component alignment, yet its capability to detect small-scale surface defects or particulate contaminants remains limited [16]. In the field of MEMS devices, Zheng et al. adopted a series of preprocessing operations, detail enhancement, grayscale conversion, image enhancement, and Canny edge extraction, together with an attribute-weighted Naïve Bayes improvement to the OTSU segmentation algorithm, achieving automatic extraction of MEMS surface cracks and quantitative measurement of crack length and width; although the approach improves crack detection accuracy to some extent, its performance is still sensitive to metal-surface reflections and texture-induced pseudo-cracks, and requires manual parameter tuning with limited robustness [17]. Amini et al. further integrated multi-view light-field imaging with traditional machine learning to construct an automatic defect recognition (ADR) system for MEMS wafer inspection, enabling classification and screening of wafer-surface defects; nevertheless, its capability to represent complex microstructures and small-object defects remains constrained by handcrafted features and fixed imaging configurations [18]. Overall, traditional image-processing and AOI/NDT methods offer advantages such as ease of implementation and high interpretability in defect scenarios with simple structure and strong contrast. However, the detection performance deteriorates markedly for MEMS devices, where defects are extremely small, show weak textures, and are strongly coupled with metallographic backgrounds. The high sensitivity to imaging conditions and feature-design choices prevents these traditional methods from meeting the stringent accuracy and robustness requirements of high-reliability micro-assembly inspection, thereby motivating researchers to explore deep learning-based small-object defect detection approaches.

Deep learning, particularly detection frameworks founded on convolutional neural networks (CNNs) and the YOLO family, has become the dominant technical paradigm for identifying small-object defects in MEMS and related micro-assembly devices. Shi et al. integrated WGAN-DIV-DC with an enhanced YOLOv5, where a generative adversarial network was used to augment scarce MEMS acoustic-film defect samples and achieved high mAP under real-time inference, effectively alleviating the issue of limited defect data in MEMS manufacturing; however, the resulting architecture is relatively complex and imposes a substantial computational burden [19]. Raveendran et al. targeted physical failure modes in MEMS substrates during fabrication by combining lightweight image preprocessing with CNN-based defect classification, but their approach largely relies on 2D visual appearance and shows limited capability to capture complex 3D structural anomalies [20]. Deng et al. developed a CNN-based online inspection system for packaging defects in MEMS pressure sensors, enabling automatic recognition of multiple types of encapsulation failures; however, its recall remains limited for extremely small cracks and localized delamination regions [21].

In the broader contexts of microelectronics and micro-assembly, Ingle et al. further proposed an integrated deep learning framework for pixel-level defect segmentation and classification on silicon wafers, but its dependence on large-scale high-quality annotations restricts its rapid adaptation to new process nodes [22]. Broader microelectronics and micro-assembly applications have also benefited from CNN-based defect detectors. Cheon et al. introduced a CNN-based wafer surface defect classification method capable of identifying “unknown defect classes,” significantly improving performance under complex defect patterns; yet the method focuses on global classification rather than fine-grained localization, restricting its effectiveness for small-object defect detection [23]. López de la Rosa et al. conducted a systematic review of machine learning and deep learning methods for SEM defect classification, highlighting improved feature representation compared to traditional image processing while noting limitations in cross-equipment robustness and interpretability [24]. Chien et al. employed deep CNN architectures for wafer surface defect classification, achieving end-to-end recognition of typical surface patterns; nonetheless, the model is sensitive to illumination variations and process-dependent layout differences, requiring retraining when deployed across new production lines [25]. Saqlain et al. proposed a deep convolutional network enhanced with resampling strategies, significantly improving minority-class recognition, although its performance still fluctuates for extremely rare classes [26].

Ling et al. proposed the lightweight TD-YOLO for targeting tiny defects on high-resolution printed circuit boards (PCBs), enhancing the feature pyramid and detection head of YOLOv5-nano while maintaining low computational cost; this demonstrates the feasibility of tiny-defect detection in industrial inspection, though its robustness under highly complex backgrounds remains a challenge [27]. Kim et al. proposed a skip-connected convolutional autoencoder for unsupervised PCB defect detection via error reconstruction, which performs well in small-sample scenarios but tends to over-reconstruct highly complex wiring patterns [28]. Feng and Cai then introduced a DDTR framework that integrates local detail cues with global dependency modeling to detect micro-defects in high-resolution PCBs, significantly enhancing tiny-defect detection but at the cost of a more complex architecture and higher inference overhead [29]. Tang et al. improved YOLOv8 by introducing the C2f_RVB module and attention-enhanced structures in RST-YOLOv8, achieving a better balance between accuracy and real-time performance for chip-surface micro-defects, though the architecture becomes more cumbersome, and deployment costs are increased [30]. Recent YOLO-based variants targeting strip-like, metallic, or industrial surface micro-defects further demonstrate that introducing deformable convolutions, lightweight pyramids, or tiny-object-specific detection heads can effectively improve recall for small objects. Overall, these studies confirm the potential of deep learning for detecting small-object defects across MEMSs, wafers, chips, and PCBs. However, most methods remain oriented toward relatively simple surface scratches, particles, or point-like defects. Limited attention has been paid to the more challenging defect patterns found in uncapped MEMS devices, specifically bond-wire-break and internal-cavity-contaminant defects, which simultaneously show weak textures, slender geometries, and strong coupling with complex metallographic backgrounds. This research gap warrants the development of a dedicated detection framework capable of addressing these highly intricate small-object defect scenarios.

Existing research on defect detection in MEMS devices has provided an essential foundation for improving the reliability of micro-assembly systems and has offered valuable insights into the recognition of complex small-scale structural defects. Microscopic metallographic images captured from uncapped MEMS devices are frequently affected by process-induced reflections, focal-plane deviations, metallic texture superposition, and microscopic noise. These imaging artifacts often result in weak textures, low contrast, and blurred boundaries within defect regions, which further complicate the discrimination of small-object defects. Automated inspection systems based on microscopic vision possess advantages such as non-contact measurement, repeatability, and seamless integration with online production. These benefits have made them an increasingly important direction for quality control in MEMS manufacturing. The accurate and efficient detection of bond-wire-break and internal-cavity-contaminant defects under complex metallographic backgrounds and micro-scale structural interference has therefore emerged as a key challenge for advancing pre-encapsulation automated inspection technologies. Motivated by this challenge, this study focuses on microscopic images of uncapped MEMS devices and develops a small-object defect detection method based on the YOLOv8s framework. By integrating the Dynamic Channel–Spatial Block, the Triplet Attention mechanism, and the small-object perception enhancement network, the proposed approach aims to enhance small-scale feature representation and improve the automation capability of pre-encapsulation MEMS inspection systems.

## 3. Materials and Methods

### 3.1. Structure of YOLO-DST

To address the imaging characteristics of MEMS devices, where bond-wire-break and internal-cavity-contaminant defects are extremely small in scale, show weak texture, and are easily affected by complex metallographic backgrounds, this study develops a MEMS small-object defect detection network, termed YOLO-DST, based on YOLOv8s. The backbone of the original YOLOv8s consists of 5 CBS (Conv–BN–SiLU) modules and 4 C2f (Cross-Stage Partial Bottleneck with Two Convolutions) modules arranged in a cascaded manner. When applied to MEMS devices with defects such as bond wire breaks and internal cavity contaminants that are small in scale, weak in texture, and fine in morphology, the conventional convolution operations exhibit limited capability to jointly model channel and spatial information, making it difficult to sufficiently extract discriminative features of small-object defects. To address this issue, Dynamic Channel–Spatial Convolution (DCSConv) is introduced into the YOLOv8s backbone, and a Dynamic Channel–Spatial Block (DCS Block) is constructed to replace the last 3 CBS + C2f combinations in the original backbone. By performing dynamic channel reconstruction and spatial reconstruction, the DCS Block enables adaptive multi-scale feature modeling, thereby enhancing the network’s ability to represent discriminative characteristics of wire-like defects such as bond-wire-break and internal-cavity-contaminant defects across different scales. In terms of the neck structure, the original YOLOv8s adopts an FPN–PAN-based feature fusion framework composed of multiple C2f and CBS modules to facilitate top down and bottom up multi-scale feature interaction and fusion. However, this structure has limited capability to suppress complex background interference in MEMS substrates. To this end, Triplet Attention is introduced after the C2f modules in the neck of YOLO-DST to emphasize defect-related cues and suppress background interference through cross-directional attention modeling. Meanwhile, an additional C2f module is incorporated into the original neck structure to further enhance multi-scale feature fusion. Furthermore, to strengthen the perception of small-object defects, a small-object perception enhancement network (SOPEN) based on a multi-scale Transformer architecture is designed. By introducing global context modeling, this block compensates for the limited capability of convolutional networks to model long-range dependency and is deeply fused with the features output from the neck to form an additional small-object detection head. As a result, the network’s responsiveness to weak-texture, small-object defects and its detection stability are significantly improved.

**Figure 1:**
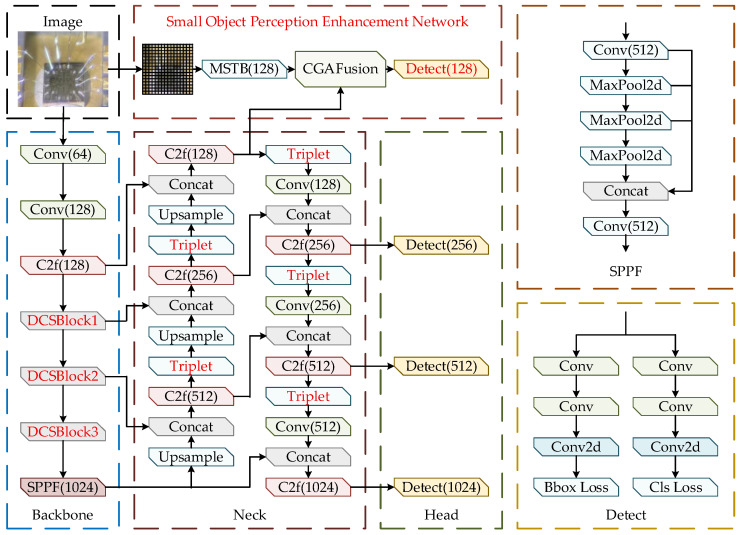
The Structure of YOLO-DST. Red blocks denote the components that differ from the original YOLOv8s, including the DCSBlock in the backbone, the Triplet Attention in the neck, and the small-object perception enhancement network and its corresponding additional small-object detection head.

The YOLO-DST network, shown in Figure 1, consists of four major components: the backbone, the neck, the detection head, and the small-object perception enhancement network. First, the metallographic image of an uncovered MEMS device is fed into the initial convolution stage and then into the backbone, where multi-scale fundamental features composed of bond wire breaks, internal cavity contaminants, and complex metallographic textures are extracted through C2f blocks and multiple DCSConv layers. The SPPF block further compresses and integrates global contextual information, producing deep semantic representations with strong discriminative ability. Next, the multi-scale features generated by the backbone are passed into the neck, where progressive feature fusion is performed through up-sampling, concatenation, and convolution operations. After each fusion stage, the Triplet Attention mechanism is applied to enhance the saliency of small-object defect regions under complex metallographic backgrounds. Meanwhile, the network incorporates a small-object perception enhancement network, which consists of an MSTB-based multi-scale Transformer, a CGA fusion block, and linear block partitioning. The block extracts fine-grained structural cues from high-resolution shallow features and fuses them with the neck features, thereby improving the detectability and stability of extremely small-object defects such as bond wire breaks and internal cavity contaminants. Finally, the fused features are fed into four detection heads with channel sizes of 128, 256, 512, and 1024 to perform classification and bounding-box regression for ultra-small, small, medium, and large defects. The smallest detection head is specifically tailored for small-object defect structures in MEMS devices, while the largest detection head strengthens contextual understanding in complex regions. By integrating the outputs of all four detection heads, the network achieves precise localization and recognition of bond-wire-break and internal-cavity-contaminant defects in uncovered MEMS devices.

### 3.2. The Dynamic Channel–Spatial Block

**Figure 2:**
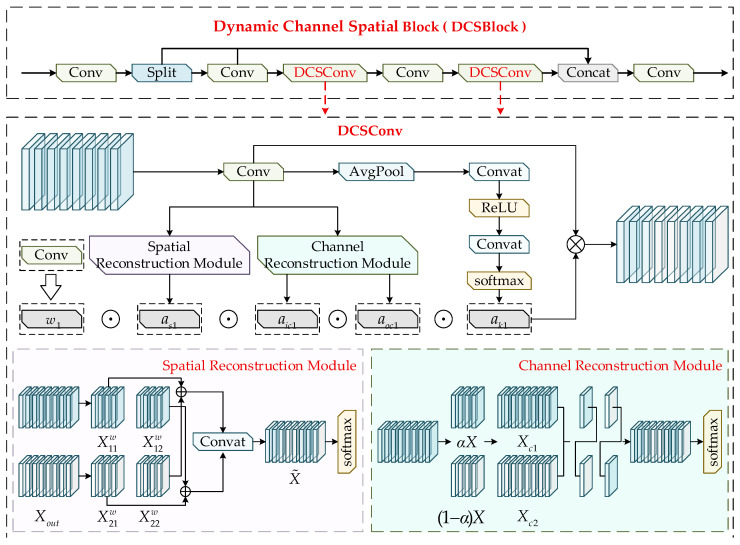
The structure of the DCSBlock.

Bond wire breaks and internal cavity contaminants in MEMS defect detection usually shows small scales, weak textures, and high similarity to complex metallographic backgrounds, which makes the modeling of local details and contextual information particularly important during feature extraction. The standard convolution in YOLOv8s is difficult to adapt to diverse minor defect features due to its reliance solely on a single static convolution kernel. Although dynamic convolution enhances feature expression ability through the combination of convolution kernels, it still cannot fully model the differences in spatial and channel dimensions, resulting in limited local feature perception ability. To alleviate this limitation in local feature modeling, this study designs DCSConv based on the characteristics of MEMS defects and replaces the conventional convolution in the C2f structure to construct the DCSBlock (Dynamic Channel–Spatial Block). This module simultaneously models feature variations along spatial and channel dimensions, significantly improving the network’s local receptive capability and fine-detail extraction, making it more suitable for identifying micro-scale defect regions. The structure of the DCSBlock is illustrated in Figure 2.

Conventional dynamic convolution computes the dynamic attention scalar $a_{k1}$ through the convolution kernels $w_{1}$ and the attention function $\pi_{wi}(x)$, but its computation fails to consider the influence of the spatial dimensions of the kernels as well as the numbers of input and output channels. Based on this, this study proposes a four-dimensional computation mechanism that incorporates the spatial kernel size k×k, the numbers of input and output channels $\left(c_{in},c_{out}\right)$, and the number of convolution kernels *n*, formulated as follows:

(1) $$y=\left(w_{1}⊙a_{s1}⊙a_{oc1}⊙a_{ic1}⊙a_{k1}\right)\cdot x,$$

In Equation (1), x and y denote the feature input and output, respectively. The scalar $a_{k1}\in R$ represents the attention weight assigned to each convolution kernel. The vector $a_{ic1}\in R^{c_{in}}$ denotes the attention weights distributed across the input channels of each convolution. The vector $a_{oc1}\in R^{c_{out}}$ represents the attention scales allocated to the output channels. The tensor $a_{s1}\in R^{k\times k}$ indicates the attention weights assigned to different spatial positions of the convolution kernel.

To reduce the redundancy introduced by conventional spatial and channel operations, this study incorporates a spatial–channel reconstruction mechanism to decrease model parameters and computational cost while enhancing feature representation capability. The structure of DCSConv consists of two components, the spatial reconstruction module and the channel reconstruction module, which are designed to eliminate redundancy during the feature extraction process.

The spatial reconstruction block aims to separate spatial redundancy from weak feature maps and evaluates the information contribution of different spatial regions using the scaling factors in Group Normalization. Given an intermediate feature map $X\in R^{N\times C\times H\times W}$ where *N* denotes the batch dimension, *C* denotes the channel dimension, and *H* and *W* represent the spatial height and width, the mean value *μ* is computed for each spatial position and normalized by the standard deviation *σ* to obtain the spatially normalized feature output as follows:

(2) $$X_{\mathrm{out}}=GN(X)=\gamma \frac{X-\mu }{\sqrt{\sigma^{2}+\varepsilon }}+\beta ,$$

Equation (2) defines *μ* and *σ* as the standard deviation and mean of spatial pixel values, respectively, while ε denotes a small positive constant. The parameters γ and β are trainable coefficients used to adjust the statistics of each batch and each channel. These parameters are introduced to enhance the spatial normalization effect and strengthen the feature modulation, formulated in Equation (3) as

(3) $$W_{ij}=\frac{\gamma_{i}}{\sum_{j=1}^{C}\gamma_{j}},\;i,j=1,2,\ldots ,C,$$

Next, the feature weight values are mapped into the interval [0, 1] through the Sigmoid function, followed by gate-controlled processing:

(4) $$W=\mathrm{Gate}\left(\mathrm{Sigmoid}\left(W_{\gamma }(GN(X))\right)\right),$$

Equation (4) sets the gating threshold to 0.5, assigning a weight of 1 when the value exceeds the threshold, and 0.5 otherwise. The feature *X* is then multiplied by two sets of gated weights to obtain the complete feature $X_{1}^{w}$ and redundant feature $X_{2}^{w}$ These two feature groups are further interactively reconstructed and concatenated to produce the refined features ${\tilde{X}}_{1}^{w}$ and ${\tilde{X}}_{2}^{w}$, along with the newly generated spatially reconstructed feature. A Softmax function is applied thereafter to extract the attention coefficient $\tilde{X}$, enabling spatial feature reconstruction, formulated in Equation (5) as

(5) $$\left\{\begin{array}{l} X_{1}^{w}=X_{11}^{w}⊕X_{12}^{w}\\ X_{2}^{w}=X_{21}^{w}⊕X_{22}^{w} \end{array}\right.\;\to \left\{\begin{array}{l} {\tilde{X}}_{1}^{w}=X_{11}^{w}⊕X_{22}^{w}\\ {\tilde{X}}_{2}^{w}=X_{21}^{w}⊕X_{12}^{w} \end{array}\right.,$$

The channel reconstruction block is designed to mitigate the redundancy introduced by standard convolution during repeated operations. A partition coefficient α∈[0,1] is first assigned to feature *X*, which is divided into αX and (1−α)X. These two parts are then processed using a k×k depthwise separable convolution and a 1 × 1 pointwise convolution (DSConv), respectively, producing two intermediate channel features, as formulated in Equation (6):

(6) $$\left\{\begin{array}{l} X_{c1}=DS(\alpha X)\\ X_{c2}=PWC((1-\alpha )X)+(1-\alpha )X \end{array}\right.,$$

Global average pooling is applied to extract global statistics from both branches, expressed as

(7) $$S_{m}=\mathrm{Pooling}(X_{m})=\frac{1}{H\times W}X_{m}(i,j),\;m=1,2,$$

Subsequently, channel attention is computed to generate feature importance coefficients $\beta_{1}$ and $\beta_{2}$, which are used to adaptively combine $X_{c1}$ and $X_{c2}$. The final reconstructed channel feature $X_{c}$ is obtained according to Equations (8) and (9):

(8) $$X_{c}=\beta_{1}X_{c1}+\beta_{2}X_{c2},$$

(9) $$\left\{\begin{array}{l} \beta_{1}=\frac{e^{s_{1}}}{e^{s_{1}}+e^{s_{2}}}\\ \beta_{2}=\frac{e^{s_{2}}}{e^{s_{1}}+e^{s_{2}}} \end{array}\right.,$$

Finally, the Softmax block extracts attention weights corresponding to channel inputs and outputs, enabling channel-wise reconstruction of the feature representation.

The backbone of YOLOv8s is primarily constructed by stacking convolutional blocks, which limits its ability to effectively distinguish targets from background regions. To address this issue, the proposed DCSConv is used to replace the original conventional convolution blocks within the backbone and is integrated with the C2f structure to form the DCS Block, thereby reducing computational cost. Afterward, a feature fusion block aggregates multi-scale feature information while enhancing the network’s capability to capture full-dimensional contextual cues, which facilitates end-to-end training and further improves the prediction accuracy of the model.

### 3.3. Small-Object Perception Enhancement Network

**Figure 3:**
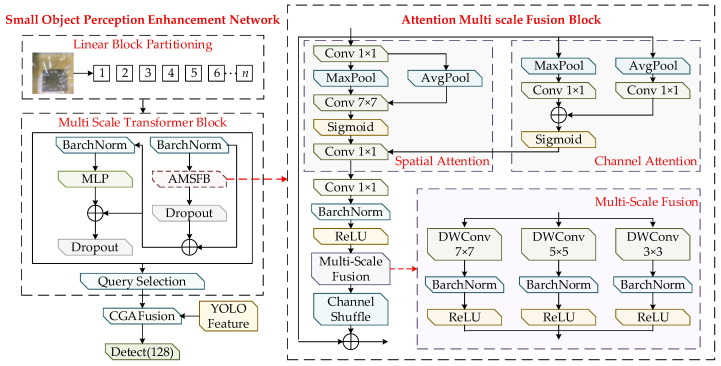
The structure of small-object perception enhancement network.

Significant differences exist in the visual characteristics of MEMS defects across different spatial scales. Large defects typically contain richer semantic structures and are more suitable for representation through high-level features, whereas small defects show extremely limited spatial extent and subtle texture details, requiring fine-grained depiction by low-level features with smaller receptive fields. If all features are extracted simultaneously at the full scale, it will not only increase the computational complexity of the network, but also may lead to feature redundancy, affecting the overall performance of the model. Considering the pronounced multi-scale properties of defects on MEMS device substrates, this study develops a small-object perception enhancement network based on the Transformer architecture [31]. The network is composed of a multi-scale Transformer block, a feature fusion module, and a small-object detection head, with the objective of strengthening the its capability to represent fine-grained defect regions. The overall structure is illustrated in Figure 3.

The extraction and fusion of small-object features begin with partitioning the down-sampled feature map into fixed-size patches, arranging them in a tiled manner, and assigning each patch a unique index. These indexed patches are then forwarded to the multi-scale Transformer block to enhance the representational capacity of the feature embeddings. The block consists of three sequential sublayers: the Attention Multi-Scale Fusion Block (AMSFB), a multilayer perceptron (MLP), and a query selection unit. Together, these components enable each token to capture not only its local pixel level characteristics but also the surrounding contextual semantics, thereby strengthening the model’s ability to perceive fine-grained defect patterns in MEMS devices.

The AMSFB comprises three components: the channel attention block (CAB), the spatial attention block (SAB), and the multi-scale fusion block (MSF). The overall design adopts a dual-path strategy. First, handle the spatial features: Reduce the channel dimension through point convolution, then extract the spatially significant regions using average pooling and Max pooling, and apply 7 × 7 convolution to the features activated by Sigmoid to generate spatial attention weights. Subsequently, process the channel features: Perform point convolution on the pooled features and generate the channel attention weights through element addition. The spatial and channel attention weights are combined through elementwise multiplication to produce refined spatial–channel feature representations. These features are further enhanced through multi-scale fusion, which integrates depthwise separable convolutions with kernel sizes of 3 × 3, 5 × 5, and 7 × 7, followed by batch normalization, ReLU activation, and summation operations. The final output is a multi-scale attention-enhanced feature representation. The AMSFB block can be formulated as follows:

(10) AMSFB(X)=MSF(SAB(X)⊗CAB(X))+X,

(11) $$CAB(X)=\mathrm{Sigmoid}(C_{1}(P_{a}(X))⊕C_{1}\;(P_{m}(X))),$$

(12) $$SAB(X)==C_{r}\;(P_{a}\;(C_{1}(X))⊕P_{m}\;C_{1}(X)),$$

(13) MSF(X)=ReLU(BN(DWC3(X)))⊕ReLU(BN(DWC5(X)))⊕ReLU(BN(DWC7(X))),

In Equations (10)–(13), CAB and SAB denote the channel attention block and the spatial attention block, while MSF represents the multi-scale fusion process. $P_{a}$ and $P_{m}$ correspond to average pooling and max pooling. $C_{1}$ and $C_{7}$ indicate the standard convolution kernels of size 1 × 1 and 7 × 7, respectively. DWConv 3 × 3, DWConv 5 × 5, and DWConv 7 × 7 refer to depthwise separable convolutions with kernel sizes of 3 × 3, 5 × 5, and 7 × 7. By introducing a multi-scale fusion mechanism into the spatial–channel attention structure, the model can more effectively capture richer feature representations while reducing the redundancy introduced by spatial–channel attention operations. As a result, the network shows improved capability to understand the global contextual information of features.

The extracted features are subsequently processed by the query-selection block, where the query key corresponds to the positions of small-object defects emphasized by the attention mechanism and the query value represents the features used to detect these defects. The module generates a sparse value-based feature map through the query-selection operation. The block takes the feature map $X_{l}$ as the input and produces the small-object defect weights $Y_{l}\in R^{H\times W}$ as the output. The size threshold $s_{l}$ is defined as the minimum regression value of the feature map $X_{l}$. Targets whose regression size is smaller than the threshold are categorized as small objects. The Euclidean distance between the target center $\left(x_{o},y_{o}\right)$ and each spatial position on the feature map is computed to encode the query-selection module. When the distance at a given position is smaller than the specified threshold $s_{l}$, that position is identified as a potential small object $k_{l}$. The module then collects all potential small objects $k_{l}$ on $X_{l}$ and extracts their corresponding feature information. However, the features of potential small objects provide only coarse estimates of the defect location. Deep features extracted from multiple convolutional stages of the YOLO model are required to refine these coarse predictions and enable accurate localization and further optimization of small-object defect detection.

To ensure consistency in spatial dimensions, bilinear interpolation is applied to align the sizes of the two types of features. Subsequently, a content-guided attention fusion mechanism (CGAFusion) [32] is employed to achieve semantic alignment across features, followed by element-wise weighted fusion, thereby providing the network with richer and more discriminative feature representations. This fused representation substantially enhances the model’s capability to describe small objects. To further improve its performance in the detection of small objects, an additional detection head with the same structure as the original ones is added to the three native detection heads in the YOLOv8s architecture. This extra head is designed to focus more effectively on small-object features, helping the model localize defects of the same category with higher confidence and reducing false detections caused by insufficient feature representation or feature confusion.

### 3.4. The Triplet Attention Mechanism

**Figure 4:**
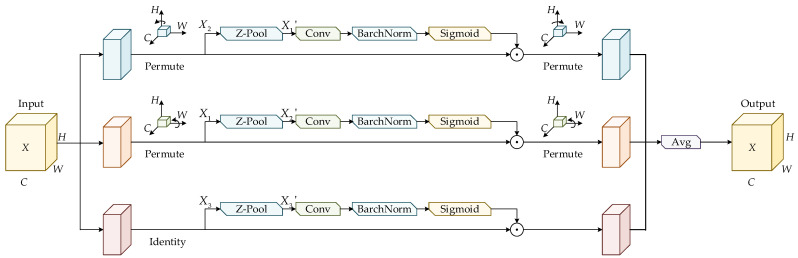
The structure of the Triplet Attention mechanism.

MEMS devices are susceptible to various background interferences during imaging, including metal reflections, etching textures, bonding residues, and particulate contamination. These factors collectively cause defects to exhibit weak textures and low contrast in the captured images, making their true information easily obscured by the background and difficult to extract effectively. To enhance the network’s sensitivity to such subtle defect patterns, the Triplet Attention mechanism is integrated into the Neck of the YOLOv8s architecture in this work. This mechanism strengthens the model’s ability to focus on micro-defect regions by improving feature representation under complex background conditions, enabling the network to concentrate more accurately on the bond-wire-break and internal-cavity-contaminant areas on MEMS substrates. As a result, the accuracy of micro-scale defect detection is significantly improved. The structure of the Triplet Attention module is illustrated in Figure 4.

The Triplet Attention mechanism assigns the input feature map $X\in R^{C\times H\times W}$ to three independent branches. In the first branch, the feature map X is rotated counterclockwise by 90° on the height axis to obtain the transformed feature map. Subsequently, the transformed feature map ${X_{1}}^{\prime }$ is processed by Z-Pool, producing an output feature map with the shape of 2×H×C. This output is further convolved and normalized before being fed into the Sigmoid activation layer to generate the attention weights. Finally, a permutation operation is applied to ensure that the output feature map remains consistent in shape with the input feature map. The computation of Z-Pool is expressed as

(14) $$\text{Z-Pool}(X)=\left[{\mathrm{MaxPool}}_{0d}(X),\;{\mathrm{AvgPool}}_{0d}(X)\right],$$

where $MaxPool_{0d}$ denotes max pooling and $AvgPool_{0d}$ denotes average pooling along the 0-th dimension. 0d indicates that both max pooling and average pooling are performed along the first dimension.

The second branch processes the input feature map through a permutation operation, rotating its spatial dimensions from H×C×W to C×H×W, which is consistent with the procedure applied in the first branch along the W dimension. The feature map X in the third branch is first passed through a channel-wise Z-Pool operation, reducing the number of channels to two and producing a simplified feature map ${X_{3}}^{\prime }$ with dimensions of 2 × *H × W*. The map ${X_{3}}^{\prime }$ is subsequently convolved and batch-normalized, and the resulting representation is fed into a Sigmoid activation layer to obtain the spatial attention weights, which are then applied to the input X.

The outputs generated by the three branches are finally aggregated by averaging, forming a refined attention feature map $X\in R^{C\times H\times W}$. The Triplet Attention mechanism constructs spatial dependencies by applying rotational operations and residual transformations to the input tensor, enabling joint encoding of channel and spatial information and improving the network’s capability to learn micro-defect patterns on MEMS device substrates.

## 4. Experiments and Results

### 4.1. Dataset

The dataset used in this experiment consists of two defect types in MEMS devices, namely bond wire breaks and internal cavity contaminants. The original MEMS metallographic images were labeled using the LabelImg tool, resulting in a dataset of approximately 10,000 MEMS defect images. After completing the annotation of the dataset, it was first divided into a training set, a validation set, and a test set in a ratio of 8:1:1. Then, various image enhancement operations, such as flipping, rotating, scaling, stitching, adding noise, shearing, translating, and Mosaic, were applied to the training set to enhance the model’s ability to adapt to different defect shapes, scale variations, and imaging differences. The validation set and the test set were not subjected to any form of enhancement operations throughout the entire experiment, to avoid potential risks of data leakage during the training, validation, and testing processes [33,34], and to ensure the objectivity and fairness of the model performance evaluation.

### 4.2. Experimental Setting

The experimental procedures of this study were conducted in a Linux operating system environment, and all deep learning parts were implemented using PyTorch 2.0. The hardware configuration consisted of a 12-core Intel Xeon Platinum 8260C processor (Intel, Santa Clara, CA, USA) and an NVIDIA GeForce RTX 3090 GPU (NVIDIA, Santa Clara, CA, USA) equipped with 24 GB of memory. The experimental code was executed in Python 3.9, with CUDA 11.7 serving as the GPU acceleration backend. The hyperparameter settings employed during model training are summarized in Table 1.

### 4.3. Evaluation Metrics

In order to comprehensively evaluate the detection effect of the proposed method on bond-wire-break and internal-cavity-contaminant defects in MEMS device metallographic images, the performance of the model was quantitatively analyzed by using indicators such as Precision, Recall, and mean Average Precision (mAP) to measure the recognition accuracy of the model on the two types of defects. In addition, the F1 score was introduced as a complementary indicator to reflect the overall balance between Precision and Recall. To evaluate the computational complexity and model size of the model, the floating-point operation count (GFLOPs) and Parameters were used to analyze the computational cost and structural complexity of the model. These metrics are defined in Equation (15).

(15) Precision==TPTP+FPRecall=TPTP+FNmAP=1N∑i=1N∫01P(R)dRF1=2×Precision×RecallPrecision+Recall,

Precision represents the proportion of samples that the model determines as positive examples and are actually positive examples. Recall represents the proportion of true positive samples that are correctly identified by the model. TP refers to the number of correctly detected positive samples, FP indicates the number of samples incorrectly predicted as positive, and FN represents the number of true positive samples that are not detected by the model. N represents the number of defect categories, and a higher mAP indicates better overall detection performance of the model. F1 is jointly influenced by Precision and Recall, and a higher value indicates a better balance between detection Precision and Recall. GFLOPs are measured in units of 10^9^ floating-point operations per second and are used to evaluate the computational complexity of the model; a larger value implies higher computational cost and greater demand on hardware resources. Parameters represent the total number of learnable parameters in the model, which is used to reflect the size and storage requirements of the model.

### 4.4. Performance Evaluation

**Figure 5:**
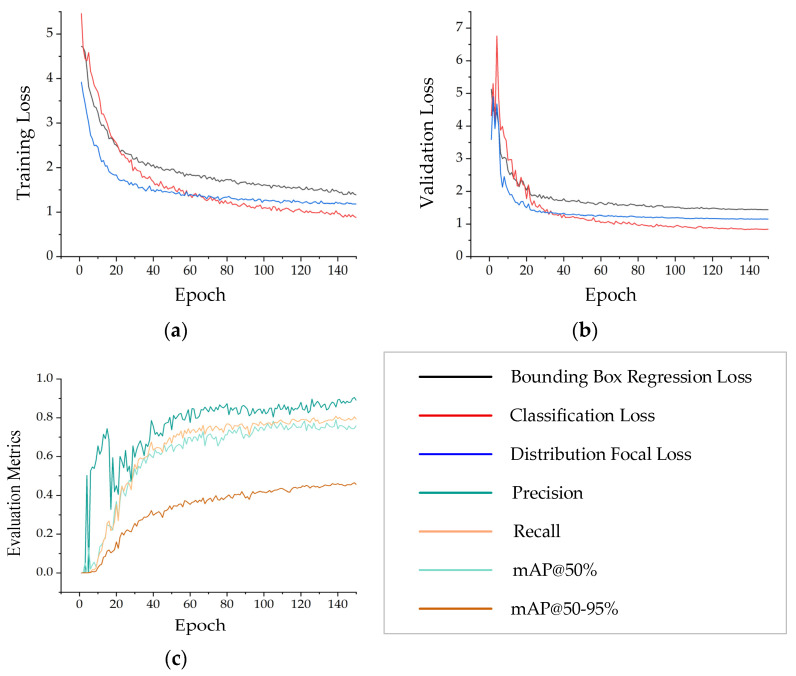
Training and validation performance curves of the YOLO-DST model: (**a**) training loss curves including bounding-box regression loss, classification loss, and Distribution Focal Loss; (**b**) validation loss curves of the same three loss components; (**c**) training evaluation metric curves including Precision, Recall, mAP@50%, and mAP@50–95%.

To evaluate the training stability and feature learning ability of the YOLO-DST model constructed in this paper in the small-object defect recognition task using uncovered MEMS devices, a metallographic image dataset containing two typical defects, namely bond wire breaks and internal cavity contaminants, was input into the model for 150 training epochs, and the loss changes and performance indicators during the training stage were simultaneously recorded. Figure 5 illustrates the trends in training loss, validation loss, and evaluation metrics such as Precision, Recall, and mAP throughout the training process. The model ultimately achieved a Precision of 90.48% and an mAP of 82.60% on the test set.

Figure 5 illustrates the trends in training loss, validation loss, and evaluation metrics for YOLO-DST. Specifically, Figure 5a displays the bounding-box regression loss, classification loss, and distribution focus loss during the training phase. All three loss metrics steadily decrease with each iteration and gradually stabilize, indicating a smooth and effective feature learning process for the model. Figure 5b presents the corresponding loss curves for the validation set. All losses show a decreasing trend and converge in a pattern consistent with the training set, indicating that the model does not suffer from significant overfitting and possesses good generalization capabilities. Figure 5c demonstrates a continuous improvement in evaluation metrics such as Recall, mAP@50%, and mAP@50–0.95% with increasing training iterations. Notably, these metrics show a steady upward trend after model convergence, indicating the network’s enhanced capability to locate and classify wire breakage and impurity defects. In Figure 5c, Precision shows a significant decline during the 18th–22nd epochs. However, it then rapidly rebounds and maintains steady and continuous growth. At this time, the model is in the transitional phase, where the intensity of the data augmentation strategy is gradually increasing. The model’s adaptation to the enhanced samples fluctuates in the short term, resulting in a temporary decline in Precision. Nevertheless, the Recall remains relatively stable during the 18th–22nd epochs. Starting from the 25th epoch, as training progresses, YOLO-DST can more effectively distinguish defect features from complex metallographic backgrounds and suppress the pseudo-response regions generated in the early stage. At the same time, Precision rebounds rapidly and grows steadily in tandem with the Recall and mAP metrics in subsequent epochs, achieving a balance between Precision and Recall. Collectively, these results demonstrate that the proposed YOLO-DST network shows stable optimization trends during training and delivers robust detection performance.

### 4.5. Ablation Experiment

To verify the effectiveness of YOLO-DST in the tasks of detecting bond-wire-break and internal-cavity-contaminant defects on MEMS devices, and to analyze the contribution of each block to the model’s performance, this paper conducted ablation experiments under the same experimental settings. Using YOLOv8s as the baseline model, a Dynamic Channel–Spatial Block, a small-object perception enhancement network, and a Triplet Attention mechanism were successively introduced to construct different block combination configurations. By comparing and analyzing indicators such as Precision, mAP, GFLOPs, and Parameters under various configurations, the system assessed the impact of each block on the detection performance and computational complexity of YOLO-DST. The results of the related ablation experiments are summarized in Table 2.

From the ablation experiment results in Table 2, it can be seen that each module has an impact on the performance and complexity of the model. The Precision of the YOLOv8s baseline model (Test 1) for the MEMS device small-object defect detection task is 87.36%, mAP@50% is 79.85%, mAP@50–95% is 46.12%, GFLOPs is 28.6 G, and Parameters value is 11.21 M. After integrating the DCSBlock (Test 2), the Precision increased to 87.91%, and mAP@50–95% changed to 45.62%, while GFLOPs changed to 27.35 G and the Parameters value reduced to10.10 M, indicating that the Dynamic Channel–Spatial Blocks not only enhance the feature representation but also reduce the computational load. After further introducing the small-object detection enhancement network (SOPEN) (Test 3), the Precision increased to 88.56%, mAP@50–95% reached 45.03%, GFLOPs rose to 33.2 G, and Parameters value increased to 13.50 M. This indicates that this block has a positive effect on MEMS small-object defect detection. After further introducing the Triplet Attention mechanism (Test 4), the model achieved 90.48% in Precision and 82.60% in mAP@50%, with mAP@50–95% reaching 45.50%. The corresponding GFLOPs and Parameters values were 36.1 G and 14.20 M. Overall, as each block is gradually added together, the detection accuracy continues to improve, while the computational complexity and model size only show a moderate increase. This indicates that YOLO-DST has achieved a relatively optimal balance between detection performance, model size, and computational complexity.

### 4.6. Comparison Experiment

**Figure 6:**
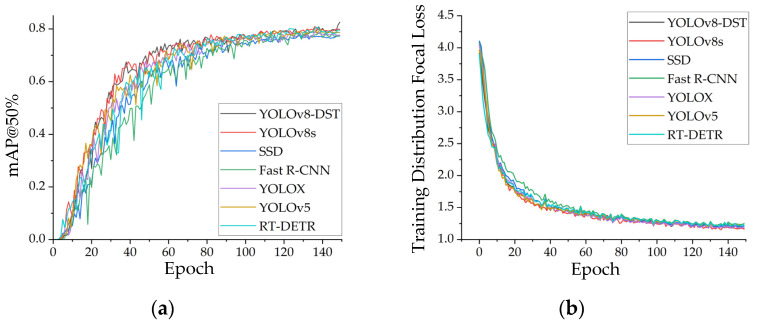
Training performance comparison of different detection models: (**a**) mAP@50% training curves of YOLO-DST, YOLOv8s, SSD, Fast R-CNN, YOLOX, YOLOv5, and RT-DETR; (**b**) training Distribution Focal Loss curves of the same models.

A comprehensive evaluation of the proposed YOLO-DST model was conducted to validate its effectiveness and performance advantages in detecting small-object defects in MEMS devices. This study selected several representative mainstream object detection models, including YOLOv8s, SSD [35], Fast R-CNN [36], YOLOX [37], YOLOv5, and RT-DETR [38], as baseline methods. All models were trained and tested under identical datasets, training strategies, and hyperparameter settings. Furthermore, key performance indicators, namely Precision, mAP, F1, and GFLOPS, were employed to assess and compare the overall performance of each method. The variations in mAP@50% and Distribution Focal Loss during the training process for different models are illustrated in Figure 6, and the quantitative comparison of performance metrics is summarized in Table 3.

The trends shown in Figure 6 indicate that all models show reasonable convergence behavior during training, yet their performance differs markedly. As illustrated in Figure 6, YOLO-DST achieves a faster rise in mAP@50% and maintains consistently higher values during the stable stage, demonstrating its stronger capability to extract discriminative features for small-object defects on MEMS devices. In contrast, SSD and Fast R-CNN show significant fluctuations, with both slower convergence and inferior final accuracy. Figure 6 further reveals that although the Distribution Focal Loss of all models decreases rapidly, YOLO-DST maintains a lower and more stable loss curve throughout training, indicating improved robustness and learning efficiency in bounding-box regression and small-object distribution modeling. Overall, YOLO-DST outperforms other mainstream detectors in terms of convergence speed, training stability, and final detection accuracy, validating the effectiveness of its architectural design for small-object defect detection.

Table 3 presents comprehensive data on the performance of different object detection models in the MEMS device small-object defect detection task under the same experimental environment settings. Overall, different detection models show significant differences in terms of detection performance and computational complexity. YOLO-DST achieves the best performance in terms of Precision (90.48%) and mAP@50% (82.60%), indicating that, compared with other baseline models, it has a significant advantage in accurately identifying MEMS small-object defects such as bond wire breaks and internal cavity contaminants. YOLO-DST achieves an F1 score of 81.96%, indicating that the model attains a reasonable balance between false positives and false negatives. Compared with the baseline model YOLOv8s, YOLO-DST achieves a noticeable improvement in detection accuracy with only a modest increase in Parameters (14.20M) and GFLOPs (36.1G), demonstrating a favorable trade-off between detection performance and model complexity. In terms of the mAP@50–95% metric, the overall performance of YOLO-DST and YOLOv8s is quite similar. The minor differences observed are mainly due to the sensitivity of this metric to the accuracy of bounding-box localization at higher IoU thresholds. The Dynamic Channel–Spatial Block, small-object perception enhancement network, and Triplet Attention introduced in YOLO-DST enhance the model’s representation capability for MEMS defects primarily from the perspectives of multi-scale feature modeling and small-object perception. These components enable the model to more effectively capture defect features at different scales while suppressing interference caused by complex metallographic textures. The design of YOLO-DST focuses on improving the completeness and stability of defect feature representations rather than explicitly optimizing bounding-box regression precision; therefore, under stricter IoU thresholds, the mAP@50–95% is slightly lower than that of YOLOv8s. In contrast, SSD and Fast R-CNN, due to their relatively traditional architectures and high computational cost, show limited detection performance and efficiency for small-object defects such as bond wire breaks and internal cavity contaminants in MEMS devices. YOLOX and RT-DETR show relatively stable performance on certain metrics but with a significant increase in computational complexity. Considering all evaluation metrics comprehensively, YOLO-DST achieves a favorable balance between detection performance, model scale, and computational complexity, demonstrating its significant advantages in detecting small-object defects such as bond wire breaks and internal cavity contaminants in MEMS devices.

**Figure 7:**
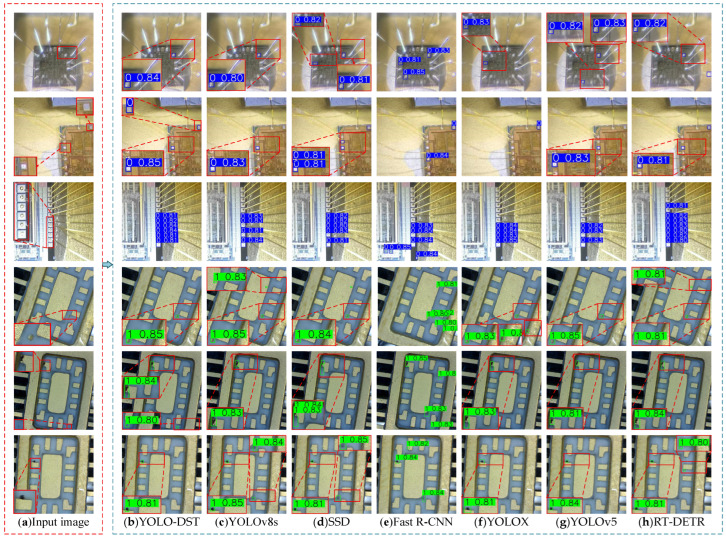
Comparative visualization of defect detection results produced by different models on MEMS devices. (**a**) Input image; (**b**) YOLO-DST; (**c**) YOLOv8s; (**d**) SSD; (**e**) Fast R-CNN; (**f**) YOLOX; (**g**) YOLOv5; (**h**) RT-DETR. In all visualizations, the label “0” denotes bond wire break, while the label “1” denotes internal cavity contaminant.

To intuitively show the performance differences among various models in detecting small-object defects in uncovered MEMS devices, and to further verify the superior effectiveness of the proposed YOLO-DST in practical MEMS defect detection tasks, this study presents a visual comparison of the detection results on the test set. By showcasing representative detection examples of bond-wire-break and internal-cavity-contaminant defects, the visualization enables a clearer assessment of each model’s capability to capture fine-grained features and accurately localize small defects under weak textures, extremely small scales, and complex metallographic backgrounds. The corresponding visualization results are shown in Figure 7.

The comparative detection results in Figure 7 directly and intuitively reflect the actual performance of different models in the MEMS device small-object defect detection task. Although only a limited number of representative detection results are presented in this study, the selected samples cover challenging scenarios with complex metallographic textures, strong illumination reflections, and blurred defect boundaries. Under these conditions, YOLO-DST is still able to stably localize small-object defects such as bond wire breaks and internal cavity contaminants, with complete bounding-box coverage and effective suppression of background interference. This performance is closely related to the incorporation of the Dynamic Channel–Spatial Block, the small-object perception enhancement network, and the Triplet Attention mechanism in YOLO-DST. In contrast, YOLOv8s and YOLOv5, although equipped with strong backbone and neck architectures, still lack fine-grained compensation for extremely small and weak-texture defects due to their standard feature pyramid designs. This often results in insufficient feature activation, leading to missed detections or boundary deviations in weak-signal regions. YOLOX, which employs a decoupled head and an anchor-free detection mechanism, improves training stability; however, due to inadequate integration of global contextual information during feature refinement, it continues to suffer from bounding-box drift. SSD and Fast R-CNN, as traditional detection frameworks, present inherent structural limitations: SSD relies on coarse multi-scale default boxes with limited resolution, making it more susceptible to missing micro-defects. Fast R-CNN depends on region proposal mechanisms, which struggle with slender and irregular defect shapes, resulting in frequent false detections or incomplete localization. RT-DETR benefits from Transformer-based global feature modeling and is capable of capturing long-range dependencies. However, the absence of dedicated small-object enhancement blocks leads to insufficient sensitivity to fine-grained structures, causing incomplete boundary detection, especially for slender wire-break defects or tiny cavity contaminants. Overall, YOLO-DST demonstrates clear superiority in terms of localization accuracy, bounding-box completeness, and robustness to weak-texture defects. These advantages stem from its architecture being specifically optimized for small-object feature representation, fully validating the effectiveness of the proposed improvements for micro-scale defect detection in MEMS devices.

## 5. Conclusions

In this paper, based on the metallographic imaging data of uncapped MEMS devices, a YOLO-DST detection model for small-object structures is proposed to address the need for automatic detection of bond wire breaks and internal cavity contaminants. Based on the YOLOv8s framework, this paper introduces the DCS Block to enhance the modeling ability of weak texture features, and integrates the Triplet Attention mechanism to suppress the interference of complex metallographic backgrounds. And the small-object perception enhancement network is integrated into the detection head, which improves the perception and localization ability of the model in extremely small-defect scenarios. Through comparative experiments with various mainstream detection models, the effectiveness of the proposed method in MEMS small-object defect detection was further verified.

- YOLO-DST combines multi-scale feature modeling, attention enhancement, and a small-object feature perception block to effectively alleviate the problem that with traditional detection methods, which can easily miss small-object defects in MEMS devices, and achieves the best performance in Precision, mAP@50%, and F1. It shows good robustness and generalization ability.
- YOLODST proposed in this paper is a defect detection method specifically designed for two small-object defects in MEMS devices: bond wire breaks and internal cavity contaminants. Although the current MEMS defect dataset covers multiple types of metallographic images, it only includes two small-target defect types, bond wire breaks and internal cavity contaminants, and the original sample quantity is limited. Therefore, the generalization ability of the model may be limited. In the future, it will need to be further verified on more defect types and larger-scale datasets to better reflect the actual application scenarios.

## Figures and Tables

**Table 1:** Training hyperparameter settings. All the experiments in this paper were conducted under the unified software and hardware configuration mentioned above to ensure the fairness and comparability of the experimental results.

Hyperparameters	Setting
Epoch	150
Batch Size	8
Image Size	640 × 640
Initial Learning Rate	0.001
Decay	0.0005
Momentum	0.9

**Table 2:** The results of the ablation experiment, where “√” indicates that the module is used and “×” indicates that the module is not used.

Test. No	YOLOv8s	DCSBlock	SOPEN	Triplet	Precision	mAP@50%	mAP@50–95%	GFLOPs	Parameters
1	√	×	×	×	87.36%	79.85%	46.12%	28.6 G	11.21 M
2	√	√	×	×	87.91%	79.73%	45.62%	27.35 G	10.10 M
3	√	√	√	×	88.56%	79.63%	45.03%	33.2 G	13.50 M
4	√	√	√	√	90.48%	82.60%	45.50%	36.1 G	14.20 M

**Table 3:** The results of the comparison experiment.

Models	Precision	mAP@50%	mAP@50–95%	F1	GFLOPs	Parameters
YOLO-DST	90.48%	82.60%	45.50%	81.96%	36.1 G	14.20 M
YOLOv8s	87.36%	79.85%	46.12%	83.18%	28.6 G	11.21 M
SSD	79.08%	77.35%	43.55%	79.65%	61.2 G	23.78 M
Fast R-CNN	78.04%	77.31%	43.19%	79.30%	370.5 G	137.3 M
YOLOX	86.35%	77.64%	45.21%	80.43%	156 G	54.32 M
YOLOv5	86.08%	78.69%	44.30%	80.60%	16 G	7.03 M
RT-DETR	89.98%	79.48%	44.56%	81.31%	56.7 G	19.87 M

## Data Availability

The original contributions presented in this study are included in the article. Further inquiries can be directed to the corresponding author.
